# Elective Tracheotomy Practices in Turkey

**DOI:** 10.1371/journal.pone.0166097

**Published:** 2016-11-15

**Authors:** Bulent Gucyetmez, Hakan Korkut Atalan, Nahit Cakar, On behalf of Turkish Tracheotomy Survey Group

**Affiliations:** 1 Department of Anesthesiology, Acıbadem University School of Medicine, Istanbul, Turkey; 2 Department of Anesthesiology, Ataşehir Memorial Hospital, Istanbul, Turkey; Azienda Ospedaliero Universitaria Careggi, ITALY

## Abstract

**Objectives:**

Elective tracheotomy (ET) procedures in intensive care units (ICU) might be different in accordance with countries and ICUs’ features. The aim of the present study was to search the epidemiology of ET procedures in Turkey.

**Methods:**

A questionnaire which consists of 43 questions was sent by e-mail to 238 ICUs which were officially recognized by The Turkish Ministry of Health. All answers were obtained between August 1, 2015 and August 31, 2015.

**Results:**

Two hundred and three ICUs (85.3%) participated in this study. 177 (87.2%) and 169 (83.4%) of ICU’s were level III and mixed ICUs respectively. Anesthesiologists were the director of 189 (93.0%) ICUs. Estimated total count of admitted, mechanically ventilated and tracheotomized patients in 2014 were 126282, 80569 (63.8%) and 8989 (7.1%) respectively. Most common indication for ET was prolonged mechanical ventilation (76.9%). The first choice for ET procedure was percutaneous in 162 (79.8%) ICUs. Griggs guide wire dilatational forceps (GWDF) technique was used as the first choice for elective percutaneous tracheotomy (EPT) by 143 (70.4%) ICUs. Most common early EPT complication was bleeding (68.0%) and late EPT complication was stenosis (35.0%). While facilitation of weaning was most important advantage (26.1%), bleeding and tracheal complications were most important disadvantages for EPT (29.1%).

**Conclusions:**

Most common indications for ET are prolonged MV and coma in Turkish ICUs. EPT is the preferred procedure for ET and GWDF is the most common technique. Bronchoscopy and USG are rarely used as a guide.

## Introduction

Elective tracheotomy (ET) is performed in the ICU for airway protection in prolonged mechanical ventilation (MV), easier tracheobronchial suction, facilitation of nursing and weaning, earlier oral nutrition and to reduce trachea-laryngeal damage [1–3]. Elective percutaneous tracheotomy (EPT) was first performed in 1985 by Ciaglia [4]. Since 1985, EPT techniques have been improved and performed at the bedside in the intensive care units (ICU) [5–8]. Although recent studies suggest EPT due to several advantages, the usage of percutaneous or surgical ET is still matter of debate [9–13]. ET procedures, indications, timing and techniques in ICU might be different in accordance with countries and ICUs’ features in Europe [2, 9, 14–20]. To our knowledge, no previous survey has been performed to investigate ET practices in Turkish ICUs. Hence, the aim of this study was to investigate ET practices in Turkey.

## Materials and Methods

### Study design

Ethical approval for this study (Ethical Committee N° ATADEK 2015/8) was provided by the Ethical Committee of Acıbadem University Hospitals (ATADEK), Istanbul, Turkey (Chairperson Prof Ismail Hakkı Ulus) on 30 July 2015. A questionnaire was designed by the Department of Anesthesiology, Acıbadem University School of Medicine. In August 2015, a questionnaire which consists of 43 questions was sent by e-mail to the directors of 238 ICUs of university, research and training, private and public hospitals which were officially recognized by The Turkish Ministry of Health. Pediatric and coronary ICUs were excluded.

### Database

Hospitals’ (type, number of beds), directors’ (specialist) and ICUs’ (name, type, number of beds, level) demographics, the estimated number of patients in ICUs per year (admitted, mechanical ventilated, tracheotomized), experience, procedures, indications, techniques, timing, complications, advantages and disadvantages of elective tracheotomy were questioned and recorded.

### Statistical analysis

Statistical analysis was performed using the Wizard Pro Version 1.7.20. All of the variables in the database were summarized using descriptive statistics. Categorical data were compared using the chi-square or Kruskall-Wallis tests. The results were expressed as the percentage (%) and median (interquartile). A type 1 error was established at 0.05.

## Results

Two hundred and three ICUs (85.3%) participated to the present study. Number of university, private, research and training and public hospitals were 71 (35.0%), 56 (27.6%), 45 (22.2%) and 31 (15.2%) respectively. General, level III and mixed ICUs were 169 (83.4%), 177 (87.2%) and 169 (83.4%) respectively. In 189 (93.0%) ICUs, the directors of ICUs were anesthesiologists. In 197 (97%) ICUs, number of bed was over 6. In 128 (63.0%) ICUs, EPT experience was more than 5 years (Table 1). Estimated total number of admitted, mechanically ventilated and tracheotomized patients in 2014 were 126282, 80569 (63.8%) and 8989 (11.1% of all mechanically ventilated patients and 7.1% of all admitted patients) respectively.

**Table 1:** ICUs’ demographics.

	n (%)
Type of hospitals	
University	71 (35.0)
Private	56 (27.6)
Research and Training	45 (22.2)
Public	31 (15.2)
Type of ICUs	
General	169 (83.4)
Surgical	12 (5.9)
Internal Medicine	8 (3.9)
Cardiovascular Surgery	6 (3.0)
Pulmonology	3 (1.4)
Neurosurgical	3 (1.4)
Neurological	2 (1.0)
Directors of ICUs	
Anesthesiologist	189 (93.0)
Internist	7 (3.5)
Pulmonologist	5 (2.5)
Neurosurgeon	1 (0.5)
Thoracic surgeon	1 (0.5)
Beds	
<6	6 (3.0)
6–10	61 (30.0)
11–20	91 (44.8)
21–30	28 (13.8)
>30	17 (8.4)
Level of ICUs	
I	2 (1.0)
II	24 (11.8)
III	177 (87.2)
Category of ICUs	
Surgical	21 (10.2)
Medical	13 (6.4)
Mixed	169 (83.4)
EPT experience, n (%)	
<1 year	15 (7.4)
1–5 year	60 (29.6)
>5 years	128 (63.0)

ICU, intensive care unit; EPT, elective percutaneous tracheotomy.

Most common indications for ET were prolonged MV and coma (156 ICUs 76.9% and 30 ICUs 14.8% respectively). In 162 (79.8%) ICUs, the first choice for ET procedure was percutaneous. ET was being performed commonly in 2^nd^ and 3^rd^ week (155 ICUs, 76.3%). In 143 (70.4%) ICUs, Griggs guide wire dilatation forceps (GWDF) was being used as the first choice of EPT technique. The number and percentage of ICUs using bronchoscopy and ultrasonography (USG) as a guide was only 49 (24.1%) and 20 (9.9%) ICUs respectively (Table 2). The usage of EPT and elective surgical tracheotomy (EST) was often being decided by the ICU team (182 ICUs, 89.7%; 173 ICUs, 85.2% respectively). While EPT was being performed in the ICU by the ICU team (184 ICUs 90.6%; 172 ICUs 84.7%), EST was being performed in operating room by ENT surgeon (124 ICUs 61.1%; 174 ICUs, 85.7%) (Table 3). While No:8.0 canula was used for male patients in 145 (71.4%) ICUs, No:7.0 canula was used for female patients in 127 (62.6%) ICUs. In 175 (86.2%) ICUs, decanulation was being done by the ICU team. And, in 125 (61.6%) ICUs, they were following-up tracheotomized patients during post-ICU period. 114 (56.2%) ICUs’ directors stated that EPT was safer than EST (Table 3).

**Table 2:** Elective tracheotomy indications, procedures, techniques and timing.

	n (%)
ET indications	
Prolonged MV	156 (76.9)
Prolonged coma	30 (14.8)
Airway protection/suction	4 (2.0)
ET procedure	
Percutaneous	162 (79.8)
Surgical	31 (15.3)
Timing for ET	
In 1^st^ week	6 (3.0)
In 2^nd^ week	77 (37.9)
In 3^rd^ week	78 (38.4)
>3^rd^ week	31 (15.3)
EPT techniques	
GWDF	143 (70.4)
CBR	16 (7.9)
Percu-Twist	9 (4.4)
Multi-dilatational	8 (3.9)
Airway management during EPT	
Removal of endotracheal tube	166 (81.8)
Laryngeal mask	19 (9.4)
The usage of bronchoscopy as a guide	
Yes	49 (24.1)
No	135 (66.5)
The usage of USG as a guide	
Yes	20 (9.9)
No	163 (80.3)

CBR, Ciaglia blue rhino; ET, elective tracheotomy; EPT, elective percutaneous tracheotomy; EST, elective surgical tracheotomy; GWDF, Griggs guide wire dilatation forceps; MV, mechanical ventilation; USG, ultrasonography.

**Table 3:** Elective tracheotomy practices and choices.

	n, (%)
Who does decide for EPT?	
ICU team	182 (89.7)
ENT	2 (1.0)
Who is performing EPT?	
ICU team	172 (84.7)
ENT	10 (4.9)
Where EPT is being performed?	
ICU	184 (90.6)
Operating room	5 (2.5)
Who does decide for EST?	
ICU team	173 (85.2)
ENT	11 (5.4)
Who is performing EST?	
ICU team	8 (3.9)
ENT	174 (85.7)
Where EST is being performed?	
ICU	65 (32.0)
Operating room	124 (61.1)
Which tracheotomy procedure?	
Obese patients	
Percutaneous	73 (36.0)
Surgical	114 (56.2)
Hemaetological disease	
Percutaneous	100 (49.3)
Surgical	84 (41.4)
Patients who has had neck surgery	
Percutaneous	18 (8.9)
Surgical	169 (83.3)
Re-tracheotomized patients	
Percutaneous	43 (21.2)
Surgical	142 (70.0)
Which ET procedure is safer?	
Percutaneous	114 (56.2)
Surgical	21 (10.3)
There is no difference	41 (20.2)

ENT, ear-nose-throat; EPT, elective percutaneous tracheotomy; ET, elective tracheotomy; EST, elective surgical tracheotomy; ICU, intensive care unit.

While most common early EPT complication was bleeding (138 ICUs, 68.0%), late EPT complication was stenosis (71 ICUs, 35.0%). Whereas facilitation of weaning was the most important advantage (53 ICUs, 26.1%), bleeding and tracheal complications were the most important disadvantages for EPT (59 ICUs, 29.1%) (Table 4). Refusal of procedure by the patient’s relatives were defined as a disadvantages by the 16 ICU directors.

**Table 4:** Elective percutaneous tracheotomy complications, advantages and disadvantages.

	n, (%)
Early EPT complications	
bleeding	138 (68.0)
dislocation	25 (12.3)
local infection	6 (3.0)
airway obstruction	4 (2.0)
Late EPT complications	
stenosis	71 (35.0)
external scar	58 (28.6)
tracheomalacia	16 (7.9)
EPT advantages	
Facilitation of weaning	53 (26.1)
Airway protection	48 (23.6)
Better comfort of patient	26 (12.8)
Easier tracheal suctioning	12 (5.9)
Reduced laryngeal complications	12 (5.9)
Reduced risk of infection	11 (5.4)
Reduction of sedation needs	10 (4.9)
Easier mouth care	7 (3.5)
EPT disadvantages	
Bleeding and tracheal complications	59 (29.1)
Refusal of procedure by the patient’s relatives	16 (7.9)
Stenosis and scar	15 (7.4)
Difficulties in patient care	14 (6.9)
Increased risk of infection	11 (5.4)
Psychological trauma	7 (3.5)
Delayed ICU discharge	5 (2.5)
Disability of speaking	5 (2.5)
Decannulation difficulties	5 (2.5)
Late term fistula	4 (2.0)
Esthetic sequelae	3 (1.5)
Risk of pneumothorax	3 (1.5)
Decreased airway humidify	2 (1.0)
Cost	2 (1.0)

EPT, elective percutaneous tracheotomy; EST, elective surgical tracheotomy.

In private and public hospitals, anesthesiologists and general ICUs were significantly higher than other hospitals (p = 0.043 p = 0.038 respectively). In public hospitals, level III ICUs (51.6%), frequency (54.8%) and experience (38.7%) of EPT and the usage of GWDF (41.9%) were significantly lower than other hospitals (p<0.001 p = 0.023 p = 0.013 and p = 0.004 respectively) (Table 5). EPT was more preferred as the first choice for ET by 81.5% of all anesthesiologists and 65.1% of them had EPT experience > 5 years (Table 6). 3% of all directors was performing early tracheotomy (in first week) in their ICUs (Table 7).

**Table 5:** Comparisons among hospitals in Turkey.

	University	Research & Training	Public	Private	*P*
n	71	45	31	56	
Anesthesiologists, n (%)	63 (88.7)	40 (88.9)	30 (96.8)	56 (100)	*0*.*043*
General ICUs, n (%)	55 (77.5)	34 (75.6)	29 (93.5)	51 (91.1)	*<0*.*038*
Level III ICUs, n (%)	71 (100)	41 (91.1)	16 (51.6)	49 (87.5)	*<0*.*001*
Admitted patients (per unit in year) ^#^	500 (300–750)	760 (450–1012)	400 (180–600)	467 (268–800)	*0*.*002*
Tracheotomized patients (per unit in year) ^#^	50 (25–75)	50 (25–76)	25 (10–50)	25 (25–50)	*<0*.*001*
Prolonged MV (ET indication) n, (%)	59 (83.1)	38 (84.4)	24 (77.4)	35 (62.5)	*0*.*002*
Prolonged coma (ET indication) n, (%)	6 (8.5)	2 (5.1)	4 (14.3)	18 (32.1)	*<0*.*001*
EST, n, (%)	10 (14.1)	4 (8.9)	11 (35.5)	6 (10.7)	*0*.*003*
EPT, n, (%)	58 (81.7)	39 (86.7)	17 (54.8)	48 (85.7)	*0*.*023*
GWDF, n (%)	50 (70.4)	36 (80.0)	13 (41.9)	44 (78.6)	*0*.*004*
EPT experience >5 years, n (%)	49 (69.0)	27 (60.0)	12 (38.7)	40 (71.4)	*0*.*013*

ET, elective tracheotomy; EPT, elective percutaneous tracheotomy; EST, elective surgical tracheotomy; GWDF, Griggs guide wire dilatational forceps; ICU, intensive care unit; MV, mechanical ventilation. Results were given as percentage and median (interquartile).

^#^, median (interquartile).

**Table 6:** EPT experience of ICU directors.

	Anesthesiologists (n = 189)	Others(n = 14)
EPT (the first choice), n (%)	154 (81.5)	8 (57.1)
EPT experience > 5 years, n (%)	123 (65.1)	5 (35.7)

EPT, elective percutaneous tracheotomy.

**Table 7:** European practices about elective tracheotomy.

	ICUs	Directors	Patients / year	Procedures	Indications	Techniques	Timing	Complications	Safer method
**Germany**^**[**^^**9**^^**]**^	455 (89)	Anesth. (45.2)	-	EPT (86.1)	-	CBR (69.4)	<3^rd^ week (68)	-	EPT (27)
**Turkey**	203 (86)	Anesth. (93)	8989 (7.1)	EPT (79.8)	Pr. MV (76.9)	GWDF (70.4)	<3^rd^ week (40.9)	Bleeding (68)	EPT (56)
**UK**^**[**^^**16**^^**]**^	178 (78)	-	-	EPT (97)	**-**	CBR (64)	-	Bleeding (70)	-
**France**^**[**^^**17**^^**]**^	152 (21.5)	-	2738 (7.2)	EST (73.5)	Pr. MV (95)	**-**	<3^rd^ week (68)	-	-
**Italy**^**[**^^**19**^^**]**^	131 (30)	-	5960 (10.4)	EPT (89)	Pr. MV (58.8)	CBR (32.8)	-	-	-
**Spain**^**[**^^**15**^^**]**^	100 (41.8)	-	-	EPT (72)	-	GWDF (33)	-	-	EPT (58.5)
**Netherland**^**[**^^**14**^^**]**^	55 (87)	Intensivist (87)	1500 (2.5)	EPT (62)	Pr. MV (95)	MD (45.4)	>2^nd^ week (29)	Bleeding (25.4)	EPT (50)
**Switzerland**^**[**^^**2**^^**]**^	48 /70)	Intensivist (50)	1256 (1.3)	EPT (57)	Pr. MV (90)	-	2^nd^ week (35)	Bleeding (40)	-
**Norway**^**[**^^**18**^^**]**^	30 (100)	Anesth. (100)	-	EPT (79)	-	MD (100)	-	-	-

CBR, Ciaglia blue rhino; EPT, elective percutaneous tracheotomy; EST, elective surgery tracheotomy; GWDF, Griggs guide wire dilatational forceps; MD, multi-dilatational; Pr. MV, prolonged mechanical ventilation. Results were given as n, percentage (%).

## Discussion

The present study is the first wide survey about ET practice in Turkey and the second large survey in Europe (Table 7). In the study, All of participating units was performing ET and they were mostly managed by anesthesiologists (93.0%) (Tables 1 and 5). Moreover, anesthesiologists were also director of all cardiovascular surgery, surgical and neurological ICUs.

We observed in the study that the most important indication for ET was prolonged MV as shown in the recent surveys (Tables 2 and 7) [2, 14, 17, 19]. EPT is a procedure which is performed more than EST in accordance with European surveys, except France [2, 9, 12, 13, 16, 17]. In recent meta-analyses, it was stated that EPT was an easy, fast, less expensive procedure but there was no difference between complications and outcomes of EPT and EST [10, 12, 13, 21, 22]. In this study, EPT was used as the first option for ET by 79.8% of all ICUs. However, we thought that EPT experience > 5 years could be a determinative factor for this choice. Because, the usage of EPT in public hospitals was the lowest when compared with others and their experience > 5 years was only 38.7% (Table 5). In recent surveys, the reluctance of ICU physicians and the lack of adequate training were some reasons why not performing EPT [2, 17, 19]. According to our results, we can conclude that EPT is much more performed by experienced anesthesiologists in Turkey (Table 6). However, the fact that they usually preferred EST as the first choice in obese, surgical and re-tracheotomized patients.

It is known that multi-dilatational technique have been still preferred as EPT technique in some countries (Table 7) [14, 18]. However, single dilatation percutaneous technique was suggested due to low complication rate and it is demonstrated that GWDF was a faster method than CBR [16, 23, 24]. Yet, Cabrini et al. showed that there was no difference between mid and long term complications of single dilatation and GWDF techniques [25]. We also found that GWDF (70.4%) was often used as EPT technique in Turkey (Table 2). We think that the reason of being the first choice of this technique may be the cost and its ease.

The timing for ET is still controversial. Blot et al. said that there was no optimal time for ET in their survey [17]. Krishnan et al. defined early tracheotomy as performing tracheotomy between 1^st^ and 7^th^ days of MV [16]. Although it was shown that there was no enough evidence for advantages of early tracheotomy in some studies, Hosokawa et al. concluded that early tracheotomy was associated with higher rate of tracheotomy, shorter ICU stay, shorter sedation of duration and lower long-term mortality rate [26–28]. In the present survey, we found that ET was commonly performed in 2^nd^ and 3^rd^ week and early tracheotomy (in 1^st^ week) was being performed by only 3% of all ICUs (Tables 2 and 7). Hence, we think that all physicians may prefer to wait for tracheotomy. Even so, in this survey, tracheotomized patients were being followed-up during post-ICU period in 125 (61.2%) ICUs.

In European surveys, it is found that the most important complications of EPT was bleeding, hypoxia and tracheal stenosis [14, 18, 19]. We also found the same early and late complications for EPT. Although the usage of bronchoscopy as a guide is associated with increased airway pressure and carbon dioxide retention due to decreased ventilation, it is reported that it is also associated with lower complications [10, 16, 29]. Rudas et al. also stated that USG guided tracheal puncture was more accurate than the landmark technique but there was no difference in complications [30]. Jackson et al. suggested that bronchoscopy should be performed by only experienced team [31]. Bronchoscopy was not used as guide for EPT in European surveys except United Kingdom and Italy [9, 14, 15]. We also found that the usage of bronchoscopy and USG were low in the study (Table 2). Although early complications may be decreased by using bronchoscopy and USG, they are not commonly used in our country yet. We think that late complications can be related with duration of cannula in situ and type, care and position of cannulas.

Dosemeci et al. demonstrated that EPT time, low pH level and high PaCO_2_ could be reduced by the usage of laryngeal mask [32]. However, they didn’t find significant difference in complications. We already observed that only 9.4% of all participant was using laryngeal mask during EPT procedure (Table 2).

It is known that there are some benefits following tracheotomy such as improved patients’ comfort, easier mobilization, reduced sedation requirement and ICU stay and increased enteral toleration [16]. In this survey, facilitation of weaning, airway protection and better comfort of patient were stated as advantages for EPT. However, it was interesting that refusal of procedure by the patient’s relatives was defined as a disadvantage (Table 4). We think that it is not a disadvantage but may be an obstacle to perform EPT procedures.

## Conclusions

ET procedures are well established in Turkish ICUs. Most common indications for ET are prolonged MV and coma in our country. Although EPT is commonly preferred procedure for ET, EST is performed in obese, surgical and re-tracheotomized patients. GWDF is the most common technique. While early ET is rarely preferred, bronchoscopy and USG are less used as a guide.

## Supporting Information

S1 Text Dataset. (XLS)

S2 Text Questionnarie. (PDF)
