# Supplementary material for: Elective Tracheotomy Practices in Turkey
Source: PLoS One. 2016 Nov 15;11(11):e0166097. doi: 10.1371/journal.pone.0166097 (PMC5113050; doi:10.1371/journal.pone.0166097)
Supplement: S2 Text — (PDF) [file pone.0166097.s002.pdf]

Questionnaire;

Prevalans, indications, timing and technics of elective tracheotomy in Turkish ICUs.

A. Participants Informations
    1. Directors
        i. Name/Surname
        ii. Speciality
        iii. E-mail
        iv. Phone number
    2. Hospital
        i. Name
        ii. Number of bed
    3. Type of Hospital
        i. University
        ii. Research and Training
        iii. Public
        iv. Private
    4. Type of ICU
        i. General
        ii. Surgical
        iii. Internal Medicine
        iv. Cardiovascular Surgery
        v. Neurosurgical
        vi. Neurological
    5. Level of ICU
        i. Level I
        ii. Level II
        iii. Level III
    6. Category of ICU
        i. Surgical
        ii. Medical
        iii. Mixed
    7. Number of ICU bed
        i. <6
        ii. 6-10
        iii. 11-20
        iv. 21-30
        v. >30
    8. Elective percutaneous tracheotomy experience
        i. <1 year
        ii. 1-5 year
        iii. >5 years

B. Participants Data

1. What is your number of admitted patients per year?
2. What is your number of mechanical ventilated patients per year?
3. What is your number of tracheotomized patients per year?
   i. <10
   ii. 11-25
   iii. 26-50
   iv. 51-75
   v. >75
4. What are your elective tracheotomy indications?
   i. Prolonged mechanical ventilation
   ii. Coma
   iii. Other
5. What is your timing for elective tracheotomy?
   i. In 1st week
   ii. In 2nd week
   iii. In 3rd week
   iv. > 3rd week
6. Which tracheotomy procedure is being performed routinely in your unit?
   i. Percutaneous
   ii. Surgical
   iii. Both
7. Do you use any guideline to perform tracheotomy?
   i. Yes
   ii. No
8. Who decides to perform elective percutaneous tracheotomy?
   i. ICU team
   ii. ENT
   iii. Surgeon
   iv. Other
9. Who performs elective percutaneous tracheotomy?
   i. ICU team
   ii. ENT
   iii. Surgeon
   iv. Other
10. Where is elective percutaneous tracheotomy performed?
    i. ICU
    ii. Operating room
11. Who decides to perform elective surgical tracheotomy?
    i. ICU team
    ii. ENT
    iii. Surgeon
    iv. Other
12. Who performs elective surgical tracheotomy?
    i. ICU team
    ii. ENT
    iii. Surgeon

iv. Other

13. Where is elective surgical tracheotomy performed?
    i. ICU
    ii. Operating room
14. Which elective percutaneous tracheotomy technic is commonly performed in your unit?
    i. GWDF
    ii. CBR
    iii. Percu-Twist
    iv. Multi-dilatational
    v. Other
15. Which airway management is used during elective percutaneous tracheotomy?
    i. Removel of endotracheal tube
    ii. Laryngeal mask
16. Do you use bronchoscopy as a guide while elective percutaneous tracheotomy is performed?
    i. Yes
    ii. No
17. Do you use ultrasonography as a guide while elective percutaneous tracheotomy is performed?
    i. Yes
    ii. No
18. What are your early complications for elective percutaneous trachetomy?
    i. Bleeding
    ii. Dislocation
    iii. Airway obstruction
    iv. Local infection
    v. Pneumothorax
    vi. Other
19. What are your late complications for elective percutaneous tracheotomy?
    i. Stenosis
    ii. External scar
    iii. Tracheomalasia
    iv. Other
20. Have you ever lost a patient while performing elective percutaneous tracheotomy?
    i. Yes
    ii. No
21. Have you ever lost a patient while performing elective surgical tracheotomy?
    i. Yes
    ii. No
22. Which severe complication have you experienced while performing elective percutaneous tracheotomy?
    i. Pneumothorax
    ii. Paratracheal placement
    iii. Severe bleeding

iv. Other

23. Which severe complication have you experienced while performing elective surgical tracheotomy?
    i. Pneumothorax
    ii. Paratracheal placement
    iii. Severe bleeding
    iv. Other
24. Have you ever failed and decided to change procedure while elective percutaneous tracheotomy is performed?
    i. Yes
    ii. No
25. Which tracheotomy procedure do you prefer in obese patients?
    i. Percutaneous
    ii. Surgical
26. Which tracheotomy procedure do you prefer in patients who has had neck surgery patients?
    i. Percutaneous
    ii. Surgical
27. Which tracheotomy procedure do you prefer in patients who has hematological disease?
    i. Percutaneous
    ii. Surgical
28. Which tracheotomy procedure do you prefer in re-tracheotomized patients?
    i. Percutaneous
    ii. Surgical
29. Which tracheotomy canula do you prefer for male patients?
    i. 8
    ii. 9
    iii. Other
30. Which tracheotomy canula do you prefer for female patients?
    i. 7
    ii. 8
    iii. Other
31. When do you change the tracheotomy canula routinely?
    i. 7th day
    ii. 14th day
    iii. 1st month
    iv. 2nd month
    v. There is no a routinely change
    vi. Other
32. Who performs decanulation?
    i. ICU team
    ii. ENT
    iii. Surgeon
    iv. Other
33. Do you follow-up tracheotomized patient in post-ICU period?
    i. Yes

ii. No

34. Which tracheotomy procedure is safer according to you?
    i. Percutaneous
    ii. Surgical
    iii. There is no difference

35. What are the advantages and disadvantages of elective percutaneous tracheotomy?

Anket;

Türk yoğun bakımlarında elektif trakeotominin prevalansı, indikasyonları, zamanlaması ve teknikleri

A. Katılımcı bilgileri
  1. Direktör
     i. Ad/Soyad
     ii. Uzmanlık alanı
     iii. E-mail
     iv. Telefon numarası
  2. Hastane
     i. Adı
     ii. Yatak sayısı
  3. Hastane tipi
     i. Üniversite
     ii. Eğitim Araştırma
     iii. Devlet
     iv. Özel
  4. Yoğun bakım tipi
     i. Genel
     ii. Cerrahi
     iii. Dahili
     iv. Kardiyovasküler cerrahi
     v. Nöroşirurji
     vi. Nöroloji
  5. Yoğun bakım derecesi
     i. Level I
     ii. Level II
     iii. Level III
  6. Yoğun bakım kategorisi
     i. Cerrahi
     ii. Medikal
     iii. Karışık
  7. Yoğun bakım yatak sayısı
     i. <6
     ii. 6-10
     iii. 11-20
     iv. 21-30
     v. >30
  8. Elektif peruktan trakeotomi deneyimi
     i. <1 yıl
     ii. 1-5 yıl
     iii. >5 yıl

B. Katılımcı verileri

1. Yıllık yoğun bakıma alınan hasta sayınız nedir?
2. Yıllık mekanik ventilasyon uygulanan hasta sayınız nedir?
3. Yıllık trakeotomize hasta sayınız nedir?
    - i. <10
    - ii. 11-25
    - iii. 26-50
    - iv. 51-75
    - v. >75
4. Elektif trakeotomi indikasyonlarınız nelerdir?
    - i. Uzamış mekanik ventilasyon
    - ii. Koma
    - iii. Diğer
5. Elektif trakeotomiyi ne zaman açıyorsunuz?
    - i. 1. hafta içinde
    - ii. 2. hafta içinde
    - iii. 3. hafta içinde
    - iv. > 3. hafta
6. Unitenizde rutin olarak uygulanan trakeotomi prosedürü nedir?
    - i. Perkütan
    - ii. Cerrahi
    - iii. İkisi de
7. Trakeotomi açarken herhangi bir klavuz kullanıyor musunuz?
    - i. Evet
    - ii. Hayır
8. Elektif perkütanöz trakeotomi açılmasına kim karar veriyor?
    - i. Yoğun bakım
    - ii. KBB
    - iii. Genel cerrah
    - iv. Diğer
9. Elektif perkütanöz trakeotomiyi kim açıyor?
    - i. Yoğun bakım
    - ii. KBB
    - iii. Genel cerrah
    - iv. Diğer
10. Elektif perkütanöz trakeotomi nerede açılıyor?
    - i. Yoğun bakım
    - ii. Ameliyathane
11. Elektif cerrahi trakeotomi açılmasına kim karar veriyor?
    - i. Yoğun bakım
    - ii. KBB
    - iii. Genel cerrah
    - iv. Diğer
12. Elektif cerrahi trakeotomiyi kim açıyor ?
    - i. Yoğun bakım
    - ii. KBB
    - iii. Genel cerrah

    iv. Diğer
13. Elektif cerrahi trakeotomi nerede açılıyor?
    i. Yoğun bakım
    ii. Ameliyathane
14. Ünitenizde hangi perkütanöz trakeotomi tekniği sıklıkla uygulanır?
    i. GWDF
    ii. CBR
    iii. Percu-Twist
    iv. Multi-dilatational
    v. Diğer
15. Elektif perkütanöz trakeotomi açılırken hangi havayolu yönetimini tercih ediyorsunuz?
    i. Endotrakeal tüp geri çekme
    ii. Larengeal maske
16. Elektif perkütanöz trakeotomi açarken klavuz olarak bronkoskopi kullanıyor musunuz?
    i. Evet
    ii. Hayır
17. Elektif perkütanöz trakeotomi açarken klavuz olarak USG kullanıyor musunuz?
    i. Evet
    ii. Hayır
18. Elektif perkütanöz trakeotomi için erken komplikasyonlarınız nelerdir?
    i. Kanama
    ii. Dislokasyon
    iii. Havayolu obstrüksiyonu
    iv. Lokal infeksiyon
    v. Pnömotoraks
    vi. Diğer
19. Elektif perkütanöz trakeotomi için geç komplikasyonlarınız nelerdir?
    i. Stenoz
    ii. Eksternal skar
    iii. Trakeomalazi
    iv. Diğer
20. Elektif perkütanöz trakeotomi uygulaması sırasında hiç hasta kaybettiniz mi?
    i. Evet
    ii. Hayır
21. Elektif cerrahi trakeotomi uygulaması sırasında hiç hasta kaybettiniz mi?
    i. Evet
    ii. Hayır
22. Elektif perkütanöz trakeotomi uygulaması sırasında hangi ciddi komplikasyon ile karşılaştınız?
23. Elektif cerrahi trakeotomi uygulaması sırasında hangi ciddi komplikasyon ile karşılaştınız?
24. Elektif perkütanzö trakeotomi uygulaması sırasında başarısız olup hiç prosedür değiştirdiniz mi?
    i. Evet
    ii. Hayır

25. Obez hastalarda hangi trakeotomi prosedürünü tercih edersiniz?
    i. Perkütanöz
    ii. Cerrahi
26. Boyun cerrahisi geçirmiş hastada hangi trakeotomi prosedürünü tercih edersiniz?
    i. Perkütanöz
    ii. Cerrahi
27. Hematolojik hastalıklarda hangi trakeotomi prosedürünü tercih edersiniz?
    i. Perkütanöz
    ii. Cerrahi
28. Re-trakeotomize hastada hangi trakeotomi prosedürünü tercih edersiniz?
    i. Perkütanöz
    ii. Cerrahi
29. Erkek hastalarda kaç numara trakeotomi kanülü kullanıyorsunuz?
    i. 8
    ii. 9
    iii. Diğer
30. Kadın hastalarda kaç numara trakeotomi kanülü kullanıyorsunuz?
    i. 7
    ii. 8
    iii. Diğer
31. Trakeotomi kanülünü rutin olarak ne zaman değiştiriyorsunuz?
    i. 7. gün
    ii. 14. gün
    iii. 1. ay
    iv. 2. ay
    v. Rutin değişiklik yapmıyoruz
    vi. Diğer
32. Dekanülasyonu kim yapıyor?
    i. Yoğun bakım
    ii. KBB
    iii. Genel Cerrahi
    iv. Diğer
33. Trakeotomize hastanızı postop. dönemde takip ediyor musunuz?
    i. Evet
    ii. Hayır
34. Hangi trakeotomi prosedürü daha güvenli?
    i. Perkütanöz
    ii. Cerrahi
    iii. Fark yok
35. Elektif perkütan trakeotominin avantajları ve dezavantajları nelerdir?
